# Differential Response to Soil Salinity in Endangered Key Tree Cactus: Implications for Survival in a Changing Climate

**DOI:** 10.1371/journal.pone.0032528

**Published:** 2012-03-05

**Authors:** Joie Goodman, Joyce Maschinski, Phillip Hughes, Joe McAuliffe, Julissa Roncal, Devon Powell, Leonel O'reilly Sternberg

**Affiliations:** 1 Fairchild Tropical Botanic Garden, Center for Tropical Plant Conservation, Coral Gables, Florida, United States of America; 2 U.S. Fish and Wildlife Service, Florida Keys National Wildlife Refuges Complex, Big Pine Key, Florida, United States of America; 3 Desert Botanical Garden, Phoenix, Arizona, United States of America; 4 UMR-DIADE, Institut de Recherche pour le Développement, Montpellier, France; 5 University of Miami, Miami, Florida, United States of America; Jyväskylä University, Finland

## Abstract

Understanding reasons for biodiversity loss is essential for developing conservation and management strategies and is becoming increasingly urgent with climate change. Growing at elevations <1.4 m in the Florida Keys, USA, the endangered Key tree cactus (*Pilosocereus robinii*) experienced 84 percent loss of total stems from 1994 to 2007. The most severe losses of 99 and 88 percent stems occurred in the largest populations in the Lower Keys, where nine storms with high wind velocities and storm surges, occurred during this period. In contrast, three populations had substantial stem proliferation. To evaluate possible mortality factors related to changes in climate or forest structure, we examined habitat variables: soil salinity, elevation, canopy cover, and habitat structure near 16 dying or dead and 18 living plants growing in the Lower Keys. Soil salinity and elevation were the preliminary factors that discriminated live and dead plants. Soil salinity was 1.5 times greater, but elevation was 12 cm higher near dead plants than near live plants. However, distribution-wide stem loss was not significantly related to salinity or elevation. Controlled salinity trials indicated that salt tolerance to levels above 40 mM NaCl was related to maternal origin. Salt sensitive plants from the Lower Keys had less stem growth, lower root:shoot ratios, lower potassium: sodium ratios and lower recovery rate, but higher δ ^13^C than a salt tolerant lineage of unknown origin. Unraveling the genetic structure of salt tolerant and salt sensitive lineages in the Florida Keys will require further genetic tests. Worldwide rare species restricted to fragmented, low-elevation island habitats, with little or no connection to higher ground will face challenges from climate change-related factors. These great conservation challenges will require traditional conservation actions and possibly managed relocation that must be informed by studies such as these.

## Introduction

During this century the effects of global climate change combined with human-induced land use change are likely to exceed the resilience of many ecosystems [1]. Landscape fragmentation [2], climate [3], disturbance intensity and frequency [4], [5] interactively impact community structure and composition. For example, altered forest structure, reduced stem densities, and changed species composition have been documented in response to hurricanes on Caribbean islands [6]. Such factors and others are likely to reduce or shift species' ranges [7], [8], [9], [10]. Indeed, Thomas et al. [11] predict 21–52 percent species-level extinctions by 2050 resulting directly from climate warming alone. Rare species are especially vulnerable as they often have narrow ecological niches, restricted ranges, low abundance, and/or occur in rare habitat types [12].

When a rare species experiences sudden population collapse, identifying the threat(s) is essential to forestall extinction and develop effective conservation and management strategies. In the late 1990 s natural area managers noticed a rapid die-off of the endangered Key tree cactus (*Pilosocereus robinii* (Lem.) Byles & G.D. Rowley) in the Florida Keys, USA. We hypothesized that factors related to recent changes in site conditions, including disturbance, soil, light, and forest structure have contributed to the recent *Pilosocereus* population crash in the lower Florida Keys. Recent high intensity and frequent hurricane storm surges coupled with sea level rise [13], [14] may have negatively affected *Pilosocereus* populations that reside <1.4 m in elevation. Although hurricanes are a major disturbance factor influencing ecosystem dynamics in the Caribbean [15] and many Caribbean species are adapted to periodic hurricanes, in the past 18 years the frequency of high intensity storm events has been especially high. Nine storms with wind velocities 40–110 knots have impacted *Pilosocereus* populations with heavy rainfall and storm surges (Table 1) [16]. For example, in 1998, Hurricane Georges created 1.8 m storm surge and 21 cm rain in the Lower Keys [17] and Hurricane Wilma created an estimated 3 m storm surge in the Middle Keys and 0.83 m storm surge in the Lower Keys with 17 cm rain in 2006 [18]. Increased rate of sea level rise may exacerbate storm surge of low-lying coastal habitats [14], [19], and may cause saltwater to infiltrate the ground water [14] and raise soil salinity. If soil salinity increases beyond the tolerance levels of *Pilosocereus*, physiological stress, mortality, or limited recolonization may result. In general, plant sensitivity to salt may vary across genotypes and may be exhibited in decreased growth, osmotic stress, physiological stress, and accumulation of ions below sub-lethal concentrations of 100 mM NaCl [20]. Changes in vegetation related to salt tolerance have already been demonstrated in the Florida Keys pine rockland ecosystems [21], [14]).

**Table 1:** Tropical storms and hurricanes affecting *Pilosocereus* populations in the Florida Keys.

Year	Storm Name	Storm Category	Wind Gust (kt)	Rainfall (cm)	Storm Surge (cm)
1994	Gordon	TS	45	2	NR
1998	Georges	H2	90	21	183
1999	Irene	H1	89	22	46
2005	Dennis	H1	80	15	51
2005	Katrina	H2	85	28	61
2005	Rita	H2	85	5	152
2005	Wilma	H3	110	5	83
2006	Ernesto	TS	40	12	NR
2008	Fay	TS	50	18	61

Note that storm category, wind gust, rainfall, and storm surge values are all specific to the lower Florida Keys. Some of the storms achieved higher values in other locations. Note that storms like Hurricane Rita and Hurricane Wilma may have been particularly damaging not only because of their wind speeds, but because little fresh water washed away salt accumulated from the storm surges after the events. TS = tropical storm; H1 = Hurricane 1 category, sustained winds 64–82 knots; H2 = Hurricane 2 category, sustained winds 83–95 knots; NR = not reported [16].

**Figure 1:**
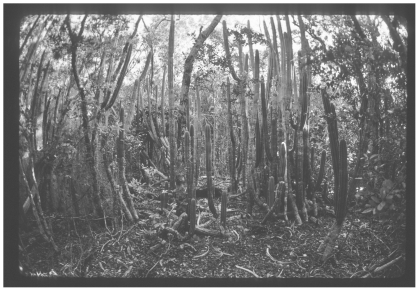
*Pilosocereus robinii* in the Lower Keys BPKW circa 1970. Dense thicket of *Pilosocereus robinii* in the Lower Keys BPKW circa 1970. Photo taken by Chris Miglicaccio.

Changes in forest structure may also be contributing to the decline of *Pilosocereus*. In forest stands in Wisconsin USA, increased canopy succession reduced the diversity of shade-intolerant understory species [22]. Historic photographs taken 40 ybp indicate that *Pilosocereus* occurred at high densities in the lower Keys preserve (Figure 1), whereas today *Pilosocereus* is rare in the diverse community that appears to have a more closed canopy. If *Pilosocereus* thrives in high light conditions associated with early sere tropical hardwood hammock, natural succession may have increased canopy closure causing *Pilosocereus* decline. Alternatively, disturbance, followed by succession may remove established community dominants and change community structure [23]. If canopy closure were negatively impacting *Pilosocereus* these conditions could potentially be manipulated by land managers to improve *Pilosocereus* health.

We compared past and current *Pilosocereus* population status and examined environmental conditions related to living and dead cacti. Based upon the findings from our observational study, we conducted a controlled greenhouse trial to test tolerance to soil salinity levels we observed in the wild sites. We used plants from two maternal lines to examine whether there was genetic variation in salinity tolerance that could help explain the differential responses observed among the wild populations. These findings prescribe the next steps for conservation of the species.

## Methods

To conduct these field and greenhouse studies, we obtained all necessary permits from US Fish and Wildlife Service (TE11069-0, TE11069-1), Florida Department of Agriculture and Consumer Services (Permits 693, 753, 756, 757,758, 759,760, 776, 823, 824,825, 830, 912, 927, 928, 943), Florida Department of Environmental Protection (Permits 5-11-08, 5-10-09, 5-09-04, 5-08-16, 5-08-38), the Village of Islamorada, and private land owners.

### Study species

**Figure 2:**
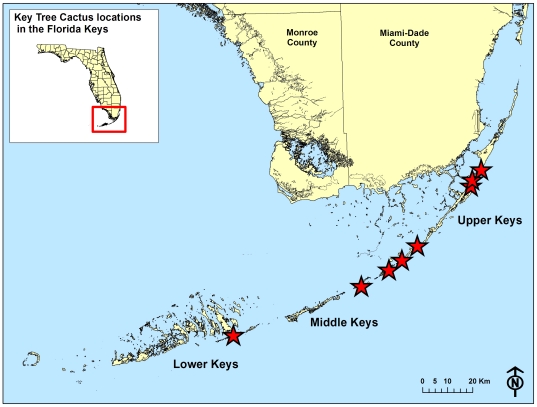
General location of *Pilosocereus robinii* populations in the Florida Keys. Map indicating general location of *Pilosocereus robinii* populations in the Florida Keys and inset of the relation of the Florida Keys to the state of Florida in USA.

*Pilosocereus robinii* occurs in the Florida Keys (Figure 2) and Cuba. This columnar cactus growing up to 10 m predominantly reproduces when wind-thrown branches produce roots and give rise to new upright stems. In contrast, seed production and dispersal are very limited; from 2007–2010 only four plants in the Florida Keys produced fruits. Since the early 1900 s habitat destruction and habitat alteration have contributed to the precarious status of the species. Great threats to the few individuals growing in few widely separated populations with low reproduction triggered the species' listing as endangered under the US Endangered Species Act in 1984 [24]. Early surveys for *P. robinii* found that the species had been extirpated from at least three Florida keys, but eight extant populations existed and one population of *P. bahamensis* (Britton) Byles & G.D.Rowley was identified [25].

Key tree cacti have unresolved taxonomy. Most recently *P. robinii* and *P. bahamensis* have been incorporated into *P. polygonus* (Lem.) Byles & G.D.Rowley [26], [27], [28], which ranges into the Bahamas, Dominican Republic, Haiti, and Cuba. However, The Flora of North America [29] states that this treatment is not supported by existing data and the Integrated Taxonomic Information System [30] treats *P. robinii* (Lem.) Byles & Rowley as a valid taxon. Herein we have followed the taxonomy accepted by U.S. Fish and Wildlife Service [24], [31] recognizing *P. robinii* and included the population of *P. bahamensis* in our surveys and monitoring.

### Study Sites

The Florida Keys are an archipelago stretching 338 km along the southwestern coast of Florida, U.S.A. between 24° and 26° N latitude and 80° and 82° W longitude (Figure 2). Though located in the subtropics, the climate is considered tropical [32] with mean temperatures of 21°C in January and 28°C in July. Average annual rainfall is 114 cm in the Upper Keys and 99 cm in the Lower Keys, based on average monthly measurements at Tavernier and Key West 1971–2000 [33]. Rainfall is seasonal with most precipitation falling between June and October [32]. From 1913 to 1990 sea level has risen at a rate of ∼2.4 cm per decade in the lower Keys [14], and recent data show the rate of sea level rise is increasing [34]. Because most areas in the Florida Keys are <2 m [32] and the highest elevation is 5.5 m, scenarios projected for sea level rise [1], [35] indicate that most of the natural areas may be eliminated within the next century [36].

*Pilosocereus* populations occur on private and public lands (Table 2). Due to the threat of illegal collection, the US Fish and Wildlife Service requested that we refer to the sites by acronyms. In the lower Keys, we conducted our in-depth ecological research at BPKW and BPKE. At all sites, *Pilosocereus* grows in dry, broad-leaved tropical hardwood hammock [37]. The substrate is typically microkarstic limestone (Holocene dolomite) with a thin (<20 cm) and variable layer of organic topsoil [14]. Tropical hardwood hammocks are diverse with more than 170 rare plants, 15 rare terrestrial animals and 12 rare birds [38], [24].

**Table 2:** *Pilosocereus* site characteristics and population numbers 1994–2011 in the Florida Keys. At US Fish and Wildlife Service request, sites are identified by acronyms only.

Site	Area (ha)	Mean elevation (m)	Stems						% change 1994–2007	% change 1994–2011	% change 2007–2011	Salinity (ppm) 2008	Salinity (ppm) 2011
			1994	2007	2008	2009	2010	2011					
BPKW	10.618	0.68±0.04	1960	27	23	14	10	10	−99	−99	−63	566	212
BPKE	2.364	1.00±0.03	240	29	25	19	17	21	−88	−91	−28	374	48
LKGOT	0.118	1.19±0.13	16	78	77	NS	87	NS	388	444	12	205	NS
LKLT	0.327	1.16±0.15	60	13	13	13	NS	18	−78	−70	38	719	38
LM* ∧	0.184	1.00±0.07	78	59	57	NS	NS	NS	−24	−27	−3	638	NS
UMCO∧	0.184	1.75±0.12	177	85	NS	50	52	43	−52	−76	−49	NS	98
UMLV	0.035	1.69±0.31	14	25	22	21	28	29	79	107	16	364	104
KL	0.026	0.52±0.14	75	112	NS	98	NS	308	49	311	175	2432	113

Elevation was calculated from LIDAR data provided by Zhang (unpublished); means ±1 S.E. are presented.

∧ Indicates privately owned land; all other sites are public lands.

* We did not receive permission to survey this private property in this year. Note that KL population is *P. bahamensis*, while the others are *P. robinii*. NS = Not Surveyed.

### Surveys and Environmental Factor Assessment

To assess population trends, we surveyed and mapped individuals within eight of nine extant populations in 2007–2011 and compared to 1994 stem counts done by Lima and Adams [25]. Because the cacti are clonal and defining individual plants was difficult, we used stems as our unit of measurement. We defined a stem as an individual trunk growing from ground level whether or not it was connected to another trunk. For all stems, we recorded condition (alive, dying or dead) and affixed a tag with a unique identification number allowing us to monitor survival over time. Dead cacti had no living tissue, but had skeletons in place. Dying cacti had yellowing or necrotic stem tissue; plants we categorized as dying in one year we found dead the next survey year.

To test which environmental factors contributed to plant mortality, in winter 2007, we measured seven environmental factors near 34 plants at BPKW and BPKE: canopy cover, average diameter and average height of nearest woody neighbor, average distance to nearest woody neighbor, tallest canopy tree height, mean elevation, and soil salinity. The total number of individuals in spatially distinct, non-overlapping locations restricted our sample size. Plants included for sampling had at least 3 m buffered spacing. At BPKW we sampled 8 living, 3 dying, and 2 dead plants and at BPKE we sampled 10 living, 9 dying, and 2 dead plants. At each individual, we characterized the surrounding vegetation structure using a modified point-centered quarter sampling method [39] with the cactus as the center of each sampling plot. In each of four quadrants delimited by the four cardinal directions, we measured the distance to the nearest living woody neighbor (NWN) >2 m tall, and recorded species, height estimated to the nearest 0.5 m, and stem diameter 20 cm above ground level. For analysis, we averaged the values from the four quadrants at each sampling point. We also recorded the species and height of the tallest canopy tree directly above the cactus. To assess canopy cover, we took photographs of the canopy from 2 m above the ground 0.5 m due south of the cactus at each sampling point. Using ImageJ, free public domain software available from the National Institute of Health [40], we converted the images so that all pixels were either categorized as canopy or sky and then computed average percent canopy cover.

We obtained elevation data from a Light Detection and Ranging (LIDAR) data set for the Florida Keys [41] and used the Spatial Analyst extension with ArcGIS [42] to derive elevation above sea level for each sampling point. The data had 5 m horizontal resolution and ±0.17 m vertical resolution at 95% confidence interval for points tested against high accuracy GPS readings on roads. We calculated average elevations of sites where plants were alive, dead and dying.

To quantify soil salinity, we collected four soil samples at approximately 10–15 cm depth adjacent to each of 34 plants at BPKE and BPKW. In addition, we collected 5–10 soil samples near *P. robinii* at BPKW, BPKE, LKLT, UMLV and KL in the winter dry seasons of 2008 and 2011 to examine temporal changes in soil salinity across the species' range. Following protocol of Rhoades [43], we measured electrical conductivity (mS/cm) of soil solutions and converted it to parts per million (ppm) dissolved salts. In 2011, we assessed sodium ion concentration in soils using a Horiba Compact Ion Meter (Horiba, LTD. Kyoto, Japan).

### Experimental Assessment of Salinity Tolerance

**Figure 3:**
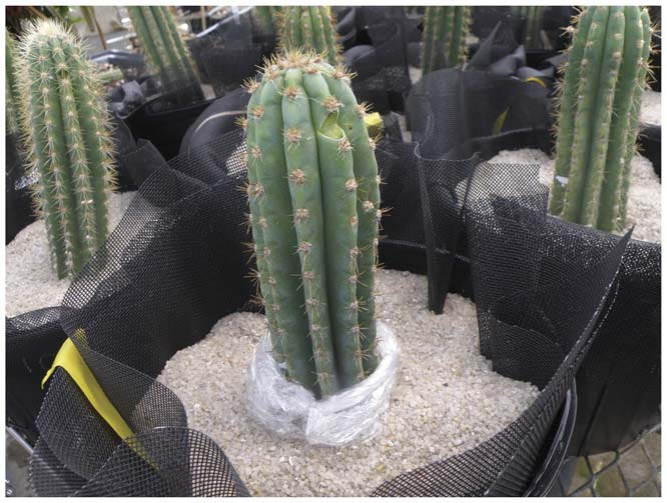
Cutting from 2 yr old seedling used in salinity experiment. Each experimental plant was the severed top of an individual seedling; seedlings arose from two maternal lines. Note tissue removed for sodium and potassium measurement appears as a notch in the stem. The plastic collar wrapped around the base was installed for easy removal of the stem from the sand prior to breaking down the experiment. It was not in place during the entire 7 weeks of the experiment. Photo taken by: Joyce Maschinski.

Based upon our findings from the above observational study, we initiated a controlled *P. robinii* salinity tolerance trial on Jan 28, 2011, in a Center for Tropical Plant Conservation greenhouse at Fairchild Tropical Botanic Garden using salinity gradients that equated to our field data and thresholds for osmotic stress described by Munns and Tester [20]. Commonly salt tolerance trials use many seeds exposed to salt solutions in sand for up to 3 weeks [20], however limitations of the numbers of seeds available to us precluded this approach. Instead, we assessed how salinity level impacted root development and clonal growth on evenly aged stem cuttings from 130 nursery grown seedlings. As noted previously, *P. robinii* has limited fruit production and predominantly has clonal reproduction through rooting of fallen branches. Population growth of *P. robinii* is largely dependent upon vegetative reproduction rather than seedling recruitment. To test how vegetative reproduction is influenced by salinity levels, we severed the top 8–10 cm of 130 stems originated from 130 seeds germinated in our nursery in 2008 from one fruit collected from each of two maternal lines; 91 cuttings came from each of 91 separate seedlings of Maternal 1, a plant cultivated in a private garden in Miami, FL originally purchased at a plant sale, and 39 cuttings came from each of 39 separate seedlings Maternal 2, formerly growing at BPKE in the lower Florida Keys (Figure 3). The maternal plant died in the wild in 2010. Thus, our stem cuttings from 130 seedlings were all of known age, all from the top of individual shoots on separate seedlings, none had roots, and all were grown under similar nursery conditions before being exposed to salinity treatments. We used stratified random sampling to assign 26 cuttings of relatively similar size classes into one of five treatment groups. We potted each cutting into a one gallon pot lined with fiberglass mesh and filled with 3.27 L of coarse sand. To avoid any drying or salt accumulation within the pots, every other day for seven weeks, we flushed each pot with 600 ml of treatment solution: 1) control plants received only reverse-osmosis water with no detectable sodum ions (0 mM NaCl); 2) 2 mM NaCl represented low soil sodium concentrations we detected at one proposed reintroduction site; 3) 15 mM NaCl represented the mean soil sodium concentration detected near live *P. robinii* in 2007; 4) 40 mM NaCl is the threshold for osmotic stress in salt-sensitive plants and comparable to the mean soil sodium concentrations measured near dead plants in 2007; and 5) 80 mM NaCl is twice the sodium concentration threshold for osmotic stress [20]. In addition all cuttings received 50 ml of 0.1% Hoagland's solution once a week for seven weeks.

To assess *P. robinii* tolerance to salinity, we measured shoot growth and changes in biomass of shoot and adventitious roots that developed from cuttings during the seven week experiment. Prior to and after the salinity treatment, we weighed and measured height of each cutting. On March 21, 2011, we severed roots from each cutting, dried them, and recorded dry mass.

To determine whether plants could recover from a seven-week exposure to salt, we assessed post-salinity treatment growth and survival. We placed each cutting with severed roots into a standard potting mix (equals parts commercial mix, sand, pea-sized rock, perlite) in a gallon pot and watered with tap water as needed every 7–10 days for 12 weeks. We recorded stem growth and survival on June 15, 2011.

Because osmotic and ionic stress may also contribute to physiological stress, we collected a small tissue sample from the meristem of all cuttings and examined δ ^13^C across salinity treatments and maternal lines. We dried samples at 50°C and placed samples weighing approximately 5 mg (±0.5 mg) in a small tin cup (8×5 mm, Elementar America, New Jersey, USA) and sealed by crumbling the tin cup into a small sphere. We placed samples in a 92 well plate (Nunc, Thermo Fisher Scientific, New York, USA) with an internally calibrated isotope standard (Soy protein, Iso-Rich Soy, Jarrow Formulas, California, USA) calibrated to one International Atomic Energy Standards (IAEA C-6 Sucrose) and two USGS standards (L-Glutamic Acid USGS41-USGS40).

We placed small spheres containing the samples and standards in the carousel of an elemental analyzer (Eurovector, Milan, Italy) connected in tandem with an Isoprime isotope ratio mass spectrometer (Elementar, Hanau, Germany). The samples were sequentially dropped via the Eurovector mechanism into an oxidizing reaction tube containing chromium oxide, and silvered cobalt oxide (Elementar America, New Jersey, USA) held at 1050°C. We added an aliquot of 10 ml of pure oxygen to increase oxidation and used ultra high purity helium as a carrier gas (AirGas, Pennsylvania, USA). We passed gases from the oxidation through a copper reaction vessel held at 650°C to reduce any nitrous oxides to nitrogen gas, a desiccant trap containing magnesium perchlorate and on through a 1.5 m molecular sieve column to separate nitrogen from carbon dioxide. The two gases were sequentially introduced into the mass spectrometer for isotopic analysis.

We calculated δ^13^C = (R_Sample_/R_Standard_ – 1)×1000, where δ^13^C represents the isotopic abundance of carbon, and R_Sample_ and R_Standard_ represent the ratio of heavy to light isotopes from the sample and standard respectively. The precision of analysis is ±0.1‰ for δ^13^C values.

To determine how salinity treatments and maternal line influenced accumulation of sodium and potassium ions within *P. robinii* tissue (Figure 3), we collected a small tissue sample from each cutting, extracted intercellular fluid, and measured sodium and potassium ion concentrations using Horiba Compact Ion Meters, (Horiba, LTD. Kyoto, Japan). Salt tolerant species may actively exclude sodium ions from roots or may regulate sodium ions through preferential osmotic accumulation of potassium ions [20], thus we reported sodium ion concentrations and ratios of potassium to sodium ion concentrations within plant tissue.

### Statistical Analysis

For the observational study, we conducted a Discriminant Function Analysis (DFA) to identify those variables that could discriminate between dead (including dying) and live cacti in 2007 [44]. We then created a set of candidate logistic regression models with the variables identified by the DFA. Using Akaike's Information Criterion (AIC), we selected the best model among the candidate set for explaining plant mortality [45]. Because our sample size was small (n = 34) relative to K (the total number of estimable parameters in the model), we calculated AIC adjusted for small sample sizes as: AICc = −2 log likelihood + 2K + 2K(K+1)/(n-K-1). We derived the −2 log likelihoods from logistic regressions of the models and calculated differences in AICc values as: AICci = AICci – minimum AICc. The best model had lowest AICci value [45].

To assess whether conditions measured in the lower Keys near live and dead plants were associated with population trends throughout the species range, we examined the relationship between percent change in the number of stems in six wild populations between 1994 and 2007 to elevation and mean soil salinity measured in 2008 using linear regression [46].

We examined growth, physiological, and plant cellular ion attributes during the salinity trial using a MANOVA, where salt treatment and accession and their interaction were main fixed effects. For each individual stem cutting derived from a single seedling, we calculated root:shoot mass and K: Na ion ratios and used log transformation before analysis. As we did with the observational study, we used DFA to reduce variables included in the model before analysis. We assessed between-groups statistics for each main effect and variable [46].

To determine whether plants could recover from salt exposure, we analyzed growth of cuttings from the salinity trials that were transferred and grown for 12 weeks under favorable conditions using repeated measures ANOVA, where prior salt treatment and maternal line were main fixed effects [46]. We assessed survival of cuttings using chi-square analysis.

## Results

### Surveys and Environmental Factor Assessment

*Pilosocereus robinii* lost 84 percent of stems between 1994 and 2007 (Table 2). Populations suffering the greatest loss were in the lower Keys, particularly at BPKW and BPKE. Formerly the largest population representing approximately 80 percent of the total known plants in the USA, BPKW had 1960 stems in the 1990 s and declined to 27 stems in 2007. Stem loss continued through 2010; only 10 stems remained – a 99% loss (Table 2). Similarly, BPKE lost 88% of stems between 1994 and 2007, however between 2007 and 2011 there has been an increase in the number of stems at BPKE. Middle Keys sites had variable change in population size; two populations declined, while LKGOT had substantial increases in the number of stems (Table 2). In the upper Keys, two populations increased (UMLV and KL), but one population decreased (UMCO). No site had seedlings and sites with population declines did not exhibit clonal growth by stem rooting.

Soil salinity and elevation were the primary factors that discriminated between dead and live plants (Wilks' Lambda F = 8.8, p<0.001; Tables 3 and 4). Mean soil salinity near dead plants (517±96 ppm) was 1.5 fold greater than salinity near live plants (385±71 ppm). Mean elevation near live plants was 12 cm lower than near dead plants (0.97±0.05 m vs.1.09±0.05 m). This difference in elevation was apparent at BPKW, where dead plants (n = 5) had 0.95±0.08 m elevation and live plants (n = 8) had mean elevation of 0.77±0.07 m, but did not significantly differ at BPKE (dead n = 11, 1.15±0.04 m and live plants 1.12±0.03 m). Other environmental variables measured did not significantly differ near dead and live plants (Table 4) and thus, our hypothesis about changes in forest structure impacting plant condition was not supported. Subsequently, only salinity and elevation were included as variables in four models, and the lowest AICc indicated that the best fitting model for plant condition was the one which included both variables (Table 2).

**Table 3:** Logistic regression models evaluated for the prediction of alive and dead/dying plants (n = 34) using the Akaike Information Criterion.

Models for plant condition	n	K	Log likelihood	AICc	Δ AICc	w*_i_*
Salinity	34	2	18.37	22.76	1.9	0.22
Elevation	34	2	21.96	26.35	5.49	0.04
Salinity+elevation*	34	3	14.06	20.86	0	0.57
Salinity+elevation+salinityxelevation	34	4	13.97	23.35	2.49	0.17

Note: AIC_c_ is Akaike's Information Criterion corrected for small sample size; K is the number of estimable parameters in the model including the intercept; Δ AICc = relative AICc for each model compared to the best-supported model; and w_i_ = Akaike weight indicating the degree of support for each model (values range from 0 to 1). Best model is indicated by asterisk.

**Table 4:** Mean values ±1 SE of seven environmental factors measured near alive and dead *Pilosocereus robinii* plants at BPKW and BPKE.

	Alive			Dead		
Percent Canopy Cover	48.94	±	4.86	48.59	±	4.12
Mean Elevation (m)	0.97	±	0.05	1.07	±	0.05
Mean Diameter (cm)	5.91	±	0.62	5.26	±	0.47
Mean Distance (cm)	130.4	±	10.96	107.27	±	8.59
Mean Height (m)	3.54	±	0.16	3.41	±	0.17
Tallest Height (m)	5.64	±	0.29	5.86	±	0.28
Soil salinity (ppm)	385	±	71	517	±	96

Elevation and soil salinity discriminated live from dead plants, but the other factors did not. Mean diameter, mean distance, and mean height refer to the nearest living woody neighbor >2 m tall from *P. robinii* plants. Further description of environmental variables can be found in text.

However, distribution-wide stem loss was not significantly related to salinity or elevation. There was a non-significant trend that soil salinity measured in 2008 was negatively correlated with the change in *P. robinii* stem numbers between 1994 and 2007 (r^2^ = 0.56, F = 5.19, p = 0.085). Some populations with high soil salinity measured in 2008 had substantial stem loss (Table 1), however *P. bahamensis* at KL had exceptionally high salinity in 2008 and tremendous stem increase between 1994 and 2007. By 2011 salinity levels decreased in all locations, but BPKW maintained the highest salinity across the sites (Table 1). There was no significant relationship between elevation and change in *P. robinii* stem numbers (r^2^ = 0.16, F = 0.77, p = 0.43).

### Experimental Assessment of Salinity Tolerance

**Figure 4:**
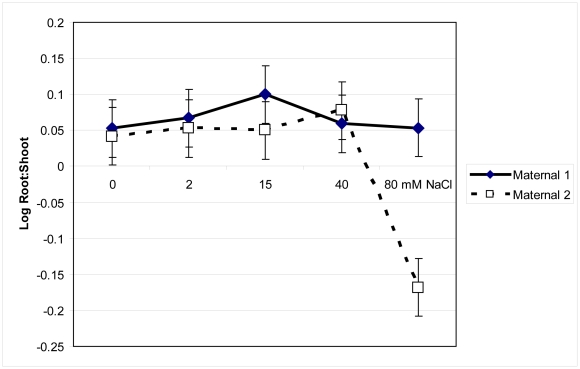
Log root:shoot mass ratios of two maternal lines of *Pilosocereus robinii*. Log root:shoot mass ratios of two maternal lines of *Pilosocereus robinii* grown for seven weeks in five salinity treatments and given 0.1% Hoagland's solution weekly. Means ±1 SE are indicated.

**Figure 5:**
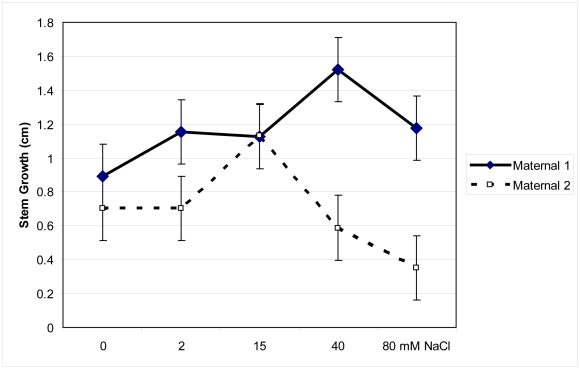
Stem growth of two maternal lines of *Pilosocereus robinii*. Stem growth of two maternal lines of *Pilosocereus robinii* cuttings grown for seven weeks in five salinity treatments and given 0.1% Hoagland's solution weekly.

*Pilosocereus* growth, physiology, and chemistry significantly distinguished salinity treatment and maternal line groups (MANOVA Wilks' Lambda F = 1.87, p<0.001). Root:shoot ratios significantly changed across salinity treatments (F = 2.93, p. = 0.02) and differed between maternal lines (F = 3.8, p. = 0.05; Figure 4). While Maternal 1 maintained root:shoot ratios across all salinity treatments, at 80 mM NaCl Maternal 2 had significantly decreased root:shoot ratio. Generally Maternal 1 had significantly greater stem growth than Maternal 2 and this difference was most pronounced at the highest salinity levels (F = 12, p = 0.0004; Figure 5).

**Figure 6:**
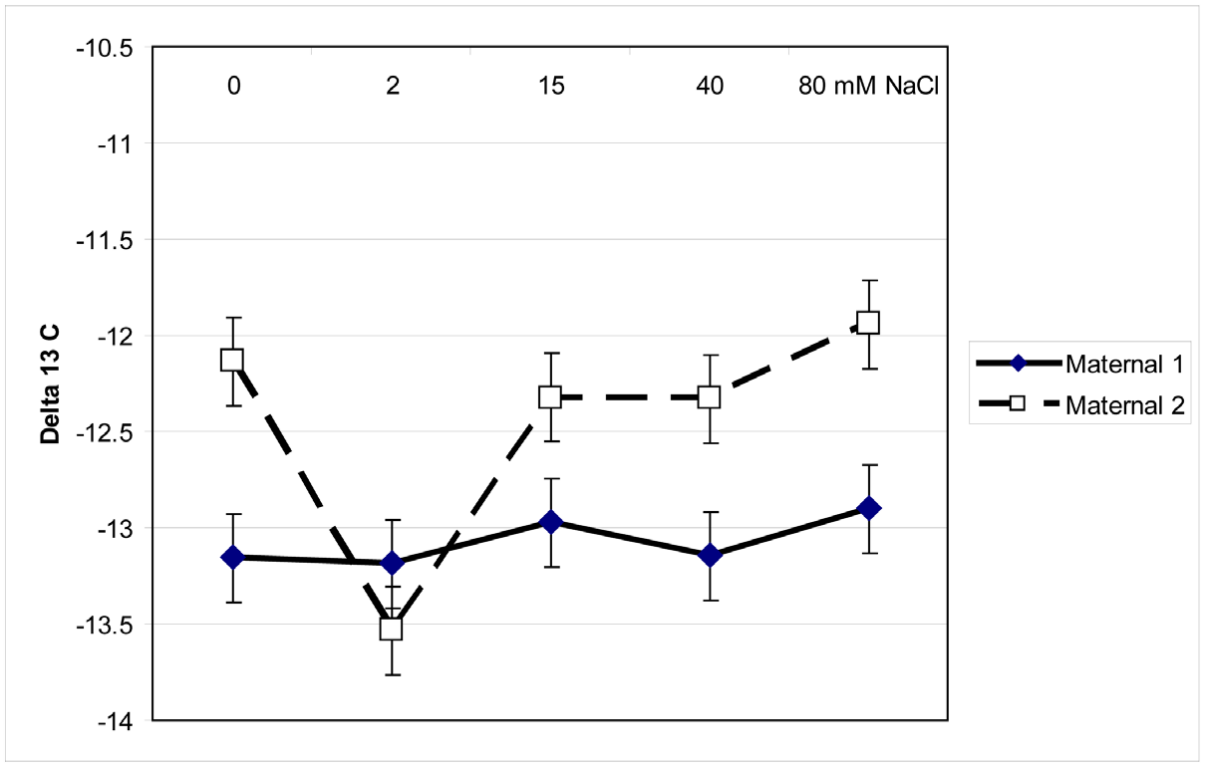
Mean δ^13^C of two maternal lines of *Pilosocereus robinii*. Mean δ^13^C of two maternal lines of *Pilosocereus robinii* rooted cuttings grown in five salinity levels for seven weeks.

Carbon isotope analysis indicated that Maternal 2 had significantly higher δ^13^C than Maternal 1 (F = 8.7, p = 0.003; Figure 6), in all but the 2 mM NaCl group, but differences across salinity treatments were not significantly different (F = 2.29, p<0.06).

**Figure 7:**
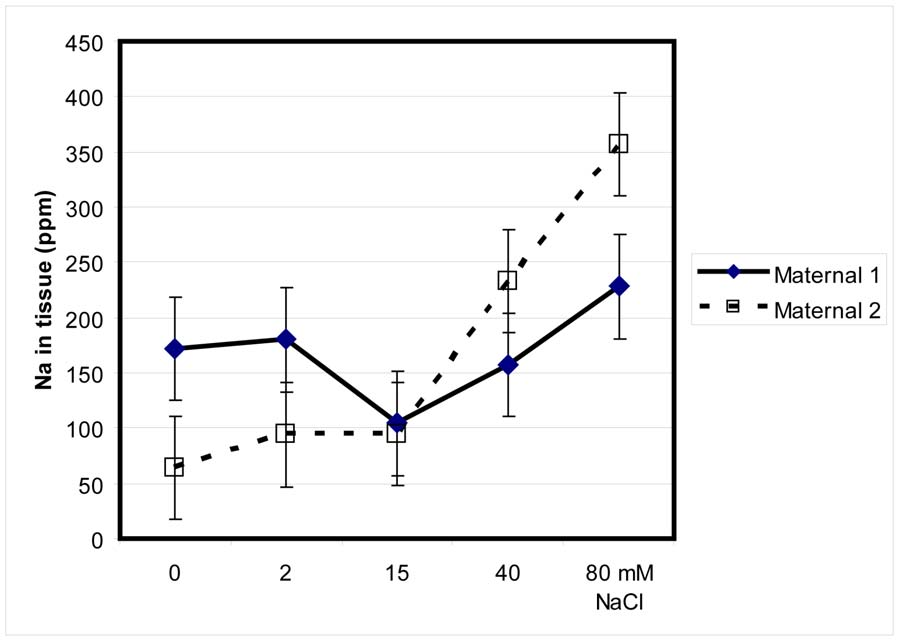
Mean sodium (Na) in plant tissue of two maternal lines of *Pilosocereus robinii*. Mean sodium (Na) in plant tissue of two maternal lines of *Pilosocereus robinii* grown in five salinity levels for seven weeks and given 0.1% Hoagland's solution weekly.

**Figure 8:**
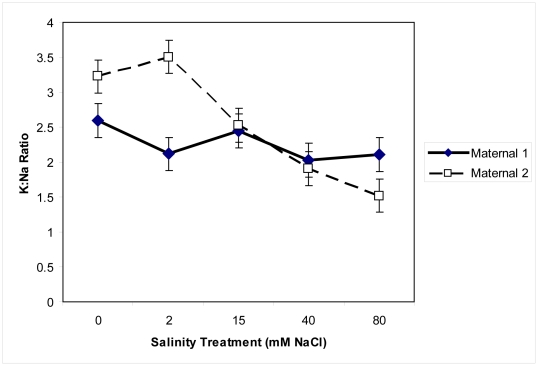
Potassium: sodium (K:Na) ratio of two maternal lines of *Pilosocereus robinii*. Potassium: sodium (K:Na) ratio in plant tissue of two maternal lines of *Pilosocereus robinii* grown in five salinity levels for seven weeks and given 0.1% Hoagland's solution weekly.

Sodium ion concentrations within plant tissue significantly differed across salinity treatments (F = 2.7, p = 0.03; Figure 7), but did not significantly differ between maternal lines (F = 0.001, p = 0.9). Sodium ions tended to accumulate in plant tissue as the concentration of salt in treatment solutions increased. This was more pronounced in Maternal 2. There was a significant interaction between maternal line and salinity treatment for potassium: sodium ions (F = 3.52, p = 0.009, Figure 8). While Maternal 1 maintained relatively stable potassium: sodium ratios across salinity treatments, Maternal 2 had high potassium: sodium ratios at the lowest salinity levels and low potassium: sodium ratios at the highest salinity levels.

**Figure 9:**
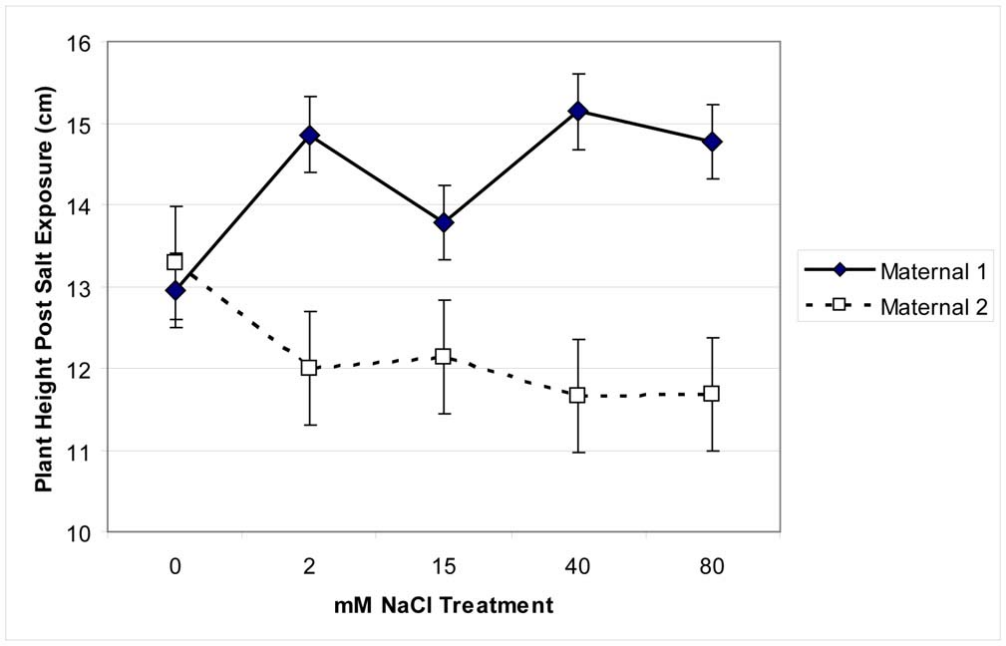
Recovery potential of *Pilosocereus robinii*. Recovery potential of *Pilosocereus robinii* as reflected by change in stem height (cm) of two maternal lines of *Pilosocereus robinii* cuttings transferred to standard potting mix and watered with tap water for twelve weeks after exposure to five salinity treatments for seven weeks.

After seven weeks of exposure to salt, most of the plants grew when returned to non-saline growing conditions, however the extent of growth depended upon maternal line and the prior salinity treatment (F = 10.01, p = 0.002; Figure 9). While Maternal 2 plants that had been exposed to 40 and 80 mM NaCl experienced less growth than controls, Maternal 1 plants that had been exposed to 40 and 80 mM NaCl had greater growth than controls.

More Maternal 1 plants survived post-salinity trials than did Maternal 2 plants, however the difference in survival was not significantly different (χ^2^ = 0.34, p>0.05). Only three plants died within 12 weeks after the salinity trial and all were Maternal 2 plants. Two were in the 40 mM NaCl group and one was in the control group.

## Discussion

Our analysis indicated that recent *Pilosocereus* decline in the Lower Florida Keys was associated with soil salinity and elevation. Soil salinity was 1.5 times greater near dying and dead plants than near live plants. However, high soil salinity was not consistently associated with the lowest elevation. It should be noted that the vertical accuracy confidence intervals of LIDAR data surpassed the differences we observed in the mean elevations of dead and live plants, therefore these findings should be interpreted with caution. It is likely that initially very subtle changes in microsite will influence soil salinity after storm events and sea level rise and will differentially impact survival or mortality of individuals along the coast.

The controlled salinity trial demonstrated that the soil salinity levels we found in the wild near dying and dead plants exceeded the physiological tolerance of Maternal 2 collected from BPK, but did not adversely affect Maternal 1. Under controlled conditions plant growth and root regeneration was inhibited at salt levels above 40 mM NaCl indicating that the BPK Maternal 2 plants are salt sensitive [20]. This is consistent with our observations that there was little rooting of new stems and high mortality at BPK in 2007–2009. Soils are considered saline at 4 dS/m, equivalent to approximately 40 mM NaCl, which is also the threshold level for osmotic stress [20]. Above this threshold growth significantly declines in many tested agricultural species [20], and this was also the case in Maternal 2. Sensitivity of Maternal 2 is possibly related to low K: Na ratio at highest salinity levels and slower growth during and after the salinity trials. The effects of exposure to salt continued for seven weeks after cuttings were transferred to standard potting mix. This suggests that following exposure to salt, effects may take weeks to manifest or it is possible that salt-sensitive genotypes will not easily recover from high salt exposure [20].

In contrast, Maternal 1 had more vigorous growth with salt treatments than when grown without salt and this effect continued even after cuttings were transferred to standard potting mix. Complex multigenic traits and a wide variety of physiological attributes may enable plant growth of Maternal 1 in the presence of salt as has been observed in other taxa [47], [48]. We measured one physiological response in Maternal 1 that was consistent with salt-tolerance: increased K: Na ratio at the highest salinity levels, suggesting selective uptake of potassium over sodium [49], [20]. There is evidence that salt-tolerance is tightly linked to succulence and crassulacean acid metabolism, as has been found in *Mesembryanthemum crystallinum* [50], [51]. Both maternal lines had δ^13^C values indicating predominantly crassulacean acid metabolism [52]. Interestingly, the more salt sensitive Maternal 2 plants showed a greater variation in δ^13^C values, greater variation in K: Na ratios, and greater variation in Na accumulation across the salinity gradient compared to values of the more salt tolerant Maternal 1 plants. Further experiments are necessary to elucidate the relationship between carbon isotope ratios, potassium accumulation, and salt tolerance. Testing Maternal 1 plant growth at even higher salinity levels would be required to determine whether it is a true halophyte [47], [48].

Our controlled salinity study may help explain reasons for differences observed in stem mortality versus stem proliferation in the Florida Keys *Pilosocereus* populations. Three *Pilosocereus* populations in the Keys (KL, LKGOT and UMLV) experienced substantial stem proliferation during 1994–2007 and 2007–2011, while other populations suffered losses in stems. At KL, *P. bahamensis* apparently tolerates very high soil salinity, as evidenced by soil salinity measurements taken in 2008 and the observed high stem proliferation. However, salt tolerance of the LKGOT and UMLV plants is untested. Our controlled salinity study suggests that origin of Maternal 1 is one of these three rapidly growing populations, but unraveling the genetic structure of salt tolerant and salt sensitive lineages in the Florida Keys will require further genetic tests. This knowledge will help elucidate conservation options.

Storm surge and sea level rise have likely contributed to high salinity levels in the soils of the Florida Keys. The transition to more salt tolerant vegetation is expected to proceed continuously from low to high elevation [14]. Thus, imperiled tropical hardwood hammocks where *Pilosocereus* grows are seriously threatened. Apparently, subtle differences in topography are influencing salt accumulation in soils and high salt levels in some locations have persisted in soils for years following storm events. Stochasticity of pulse disturbances, direction and speed of winds and storm surge will likely generate patchy mortality on a small scale, but these interact with ramp disturbances to accelerate the impacts on coastal vegetation [14]. Despite the fact that the last storm event in the lower Keys with storm surge was Aug 2008, the highest soil salinity across sites is at BPKW. This indicates that soils are probably influenced by intrusion of salt water into the inland portions of the hammock. Within coastal hammocks, rising sea level can shrink the vadose zone - the unsaturated areas in soil where fresh water from precipitation is stored, and infiltrate the fresh water lens increasing the salinity of water available to plants [53]. As sea level rises considerably further, all *Pilosocereus* populations and tropical hardwood hammock ecosystems will be impacted and salt tolerance will be essential for persistence in the Keys. Our study suggests that susceptibility to high soil salinity is more related to lineage than elevation or location of population. Results of a more extensive and ongoing genetic analysis will reveal population genetic structure in *Pilosocereus* and may reveal genetic differentiation in salt tolerance across existing wild populations.

The impacts of tropical storm activity and sea level rise along coastlines worldwide are not likely to diminish, but rather there is concern that they will continue to increase as a result of global warming [54]. Sea level rise and increased storm intensity threaten long-term persistence of *Pilosocereus* in the Florida Keys. Predicted sea level rise estimates of >1 m by the end of the next century [55], [56] would leave most *Pilosocereus* habitat under water and threaten low elevation coastal habitats worldwide. Sea level rise and warmer sea-surface temperatures create conditions that increase the frequency of intense hurricanes [54], which in turn may increase storm surge [57]. In some places plant communities may be able to shift upland with sea level rise, however coastal development and habitat fragmentation pose challenges to plant migration [58], [1]. Much depends upon the pace of the change [59]. Some species will prove to be more mobile than others, however many species will be unable to migrate fast enough to keep pace with predicted rates of change [11], [60], [61]. *Pilosocereus* has low ability to disperse the distances required to colonize suitable habitat as the areas it now occupies are overtaken by sea level rise.

To reduce extinction risk, short-term actions (within the next 1–25 years) can be integrated with the predicted long-term (∼100 years) loss of habitat from sea level rise [62]. Short-term actions include continued annual monitoring to determine whether populations will continue to decline or will recover from disturbance events [31] and capturing diverse genetic representation in ex situ holdings [63]. *Ex situ* conservation collections of seeds and/or whole plants are currently being held at our institutions. Genetic studies will be necessary to assess the appropriate source material required for experimental reintroductions within the species range. Experimental reintroductions to test environmental attributes associated with plant growth and survival at new locations are in planning stages. Based upon similarities of community structure, soil conditions, and elevation, we have identified two suitable reintroduction sites within the species' historic range [64]. Establishing new populations within range would: 1) increase total populations in the wild; 2) disperse the risk that a single hurricane could decimate all naturally occurring populations; and 3) improve our understanding of the plant's biology and habitat-specific demography [63], [65], but because all habitat within the species' current U.S.A. range has high risk from sea level rise and storm surge, this is certainly only a short-term solution [62].

Global change is presenting unprecedented opportunities for conservation across borders within the climate envelope of particular species [66]. Conservation practice would be enhanced by international intellectual exchange between the U.S.A. and Cuba regarding the species' biology, including investigations of population genetic structure, taxonomy, and demography.

If they are to persist in the future, plants endemic to coastal and island systems will likely require managed relocation to higher ground into suitable habitat within an appropriate climate envelope [62]. Species, such as *P. robinii*, with little habitat and low dispersal ability, are prime candidates for experimental reintroductions and managed relocation [67]. Islands like the Florida Keys with very little topographic relief are predicted to lose all land area to sea level rise within 100 years, such that there will literally be nowhere for species to go without moving to other land masses [62]. Some Caribbean islands have places where elevation exceeds 5 m above sea level, but whether these locations would be suitable recipient sites from a biological or political perspective is uncertain. Introducing endangered species into areas outside of their historic range is a controversial issue [68], but one that must be addressed if we are to maintain biodiversity in the face of climate change.
